# Supplementary material for: Learning from the Implementation of the Child Nutrition Program: A Mixed Methods Evaluation of Process
Source: Children (Basel). 2022 Dec 14;9(12):1965. doi: 10.3390/children9121965 (PMC9777218; doi:10.3390/children9121965)
Supplement: Supplementary file 1 [file children-09-01965-s001.zip › children-2032897-supplementary.pdf]

Supplement S1: TREND Statement Checklist

Paper Section/ Topic	Item No	Descriptor	Reported? ✓	Pg #
**Title and Abstract**				
Title and Abstract	1	• Information on how unit were allocated to interventions	✓	1
		• Structured abstract recommended	✓	1
		• Information on target population or study sample	✓	1
**Introduction**				
Background	2	• Scientific background and explanation of rationale	✓	3
		• Theories used in designing behavioral interventions	✓	3
**Methods**				
Participants	3	• Eligibility criteria for participants, including criteria at different levels in recruitment/sampling plan (e.g., cities, clinics, subjects)	✓	5
		• Method of recruitment (e.g., referral, self-selection), including the sampling method if a systematic sampling plan was implemented	✓	5
		• Recruitment setting	✓	5
		• Settings and locations where the data were collected	✓	5
Interventions	4	• Details of the interventions intended for each study condition and how and when they were actually administered, specifically including:	✓	7
		○ Content: what was given?	✓	7
		○ Delivery method: how was the content given?	✓	7
		○ Unit of delivery: how were the subjects grouped during delivery?	✓	7
		○ Deliverer: who delivered the intervention?	✓	7
		○ Setting: where was the intervention delivered?	✓	5
		○ Exposure quantity and duration: how many sessions or episodes or events were intended to be delivered? How long were they intended to last?	✓	7
		○ Time span: how long was it intended to take to deliver the intervention to each unit?	✓	7
		○ Activities to increase compliance or adherence (e.g., incentives)	✓	7
Objectives	5	• Specific objectives and hypotheses	✓	4
Outcomes	6	• Clearly defined primary and secondary outcome measures	✓	7
		• Methods used to collect data and any methods used to enhance the quality of measurements	✓	7
		• Information on validated instruments such as psychometric and biometric properties	✓	8
Sample Size	7	• How sample size was determined and, when applicable, explanation of any interim analyses and stopping rules	✓	7
Assignment Method	8	• Unit of assignment (the unit being assigned to study condition, e.g., individual, group, community)	✓	7
		• Method used to assign units to study conditions, including details of any restriction (e.g., blocking, stratification, minimization)	✓	7
		• Inclusion of aspects employed to help minimize potential bias induced due to non-randomization (e.g., matching)	✓	7

Supplement S1: TREND Statement Checklist

Blinding (masking)	9	• Whether or not participants, those administering the interventions, and those assessing the outcomes were blinded to study condition assignment; if so, statement regarding how the blinding was accomplished and how it was assessed.	✓	7
Unit of Analysis	10	• Description of the smallest unit that is being analyzed to assess intervention effects (e.g., individual, group, or community)	✓	5
		• If the unit of analysis differs from the unit of assignment, the analytical method used to account for this (e.g., adjusting the standard error estimates by the design effect or using multilevel analysis)	✓	7
Statistical Methods	11	• Statistical methods used to compare study groups for primary methods outcome(s), including complex methods of correlated data	✓	7
		• Statistical methods used for additional analyses, such as a subgroup analyses and adjusted analysis	✓	7
		• Methods for imputing missing data, if used		N/A
		• Statistical software or programs used	✓	7, 8
**Results**				
Participant flow	12	• Flow of participants through each stage of the study: enrollment, assignment, allocation, and intervention exposure, follow-up, analysis (a diagram is strongly recommended)	✓	9
		o Enrollment: the numbers of participants screened for eligibility, found to be eligible or not eligible, declined to be enrolled, and enrolled in the study	✓	12-14
		o Assignment: the numbers of participants assigned to a study condition	✓	12-14
		o Allocation and intervention exposure: the number of participants assigned to each study condition and the number of participants who received each intervention	✓	12-14
		o Follow-up: the number of participants who completed the follow-up or did not complete the follow-up (i.e., lost to follow-up), by study condition	✓	12-14
		o Analysis: the number of participants included in or excluded from the main analysis, by study condition	✓	12-14
		• Description of protocol deviations from study as planned, along with reasons	✓	26
Recruitment	13	• Dates defining the periods of recruitment and follow-up	✓	7
Baseline Data	14	• Baseline demographic and clinical characteristics of participants in each study condition	✓	9-23
		• Baseline characteristics for each study condition relevant to specific disease prevention research	✓	9-23
		• Baseline comparisons of those lost to follow-up and those retained, overall and by study condition	✓	9-23
		• Comparison between study population at baseline and target population of interest	✓	9-23
Baseline equivalence	15	• Data on study group equivalence at baseline and statistical methods used to control for baseline differences	✓	9-23

Supplement S1: TREND Statement Checklist

Numbers analyzed	16	• Number of participants (denominator) included in each analysis for each study condition, particularly when the denominators change for different outcomes; statement of the results in absolute numbers when feasible	✓	9-23
		• Indication of whether the analysis strategy was "intention to treat" or, if not, description of how non-compliers were treated in the analyses		N/A
Outcomes and estimation	17	• For each primary and secondary outcome, a summary of results for each estimation study condition, and the estimated effect size and a confidence interval to indicate the precision	✓	9-23
		• Inclusion of null and negative findings	✓	9-23
		• Inclusion of results from testing pre-specified causal pathways through which the intervention was intended to operate, if any	✓	9-23
Ancillary analyses	18	• Summary of other analyses performed, including subgroup or restricted analyses, indicating which are pre-specified or exploratory	✓	9-23
Adverse events	19	• Summary of all important adverse events or unintended effects in each study condition (including summary measures, effect size estimates, and confidence intervals)	✓	26
**DISCUSSION**				
Interpretation	20	• Interpretation of the results, taking into account study hypotheses, sources of potential bias, imprecision of measures, multiplicative analyses, and other limitations or weaknesses of the study	✓	24
		• Discussion of results taking into account the mechanism by which the intervention was intended to work (causal pathways) or alternative mechanisms or explanations	✓	24
		• Discussion of the success of and barriers to implementing the intervention, fidelity of implementation	✓	25
		• Discussion of research, programmatic, or policy implications	✓	25
Generalizability	21	• Generalizability (external validity) of the trial findings, taking into account the study population, the characteristics of the intervention, length of follow-up, incentives, compliance rates, specific sites/settings involved in the study, and other contextual issues	✓	24-25
Overall Evidence	22	• General interpretation of the results in the context of current evidence and current theory	✓	27

From: Des Jarlais, D. C., Lyles, C., Crepaz, N., & the Trend Group (2004). Improving the reporting quality of nonrandomized evaluations of behavioral and public health interventions: The TREND statement. *American Journal of Public Health*, 94, 361-366. For more information, visit: http://www.cdc.gov/trendstatement/

Supplement S2: Interview Question Outline

Site Key Informant Interview Guide- Site Level

Name of Interviewer:	Date of Interview:
Person being interviewed:	Position:
Site:	

Intro

Explain purpose, what will happen with info

Consent

Guiding Questions

1. Please can you describe your role to me? Prompt: What are your job functions at this site?

2. How long has your site been participating in the Child Nutrition Program?

3. Can you describe the how CNP operates at your site?

Program Implementation

4. Please can you describe to me how you are involved in the program implementation of CNP? (Prompt: List any management roles, activities or other actions you are a part of; Management, activities, actions)

5. Have you participated in trainings/ learning activities offered by CNP? If so, which type?

6. What were your overall impression of these trainings/services? What was successful? What challenges did you experience?

7. Have you changed the way you perform your job duties after attending the CNP Training? If so, what specifically has changed?

8. Has your site implemented any of the practices learned in the training? If yes, what practices or tools?

9. What are some practices that your site has found challenging to implement or don't use? Why were they challenging or not useful?

Screening, Tracking Progress (Database), Interventions

10. Please can you describe to me the current processes used at your organization to track and screen all children, including children with disabilities and special needs?

    a. Does your organization use the database to track children's progress? If so, are there specific people designated to use and manage this data?

11. What progress have you seen in child nutrition and growth monitoring at your site since CNP began? Please give examples. Is this what you expected? Any lessons learned?

Relevance of Project to Children's Needs

12. Can you explain how the program meets the needs of children? (If it does not meet the needs of children, can you explain why?) Is the program design relevant to the current situation of children? If not, what is challenge?

13. Does CNP fit with, or meet, the priorities of your organization/government?

14. Have you seen any changes in children's health since implementing practices from the Child Nutrition Program? If yes, what changes? If no, what do you think may help you to see changes?

Successes and Challenges

15. In your opinion, do you think the program implementation gone as planned? What have you planned to by this point that you have not been able to do?

16. What were the biggest successes of the project? (Skip questions if previously covered)

17. What are the biggest challenges?

18. I would like to discuss with you now about working with Holt – Can you describe your experience working with Holt. What has worked well in your opinion? and what could be better?

Other impacts

19. Has CNP been sustainable at your site? Have any of its practices become standard across your organization? If so, explain.

20. What additional resources might you need to sustain the CNP at your site long-term?

21. How have you adapted the Child Nutrition Program to be relevant at your site?

22. Have you used anything you learned from the Child Nutrition Program in your own home or in your community?

Recommendations

23. Do you have future recommendations for the program? Advice for future sites interested in taking on the program?

24. What are some ways that the program could better support your organization? Has the support been adequate? (Financial, technical, training)

25. Would you recommend this program to other sites? Why or why not?

26. What do you think the Child Nutrition Program would need to do to grow within the country where you work? What do you think it would take to get there?

Closing

27. Is there anything else you would like to tell me about the Child Nutrition Program?

28. Is there other information you think is important for us to know?

Thank you for your time. You can reach out to me at any point with any additional information.

Site Key Informant Interview Guide- Country Level

Name of Interviewer:	Date of Interview:
Person being interviewed:	Position:
Site:	

Intro

Explain purpose, what will happen with info

Consent

Guiding Questions

1. Please can you describe your role and job functions.

2. How long have you been a Child Nutrition Program implementor/ Champion?

3. Describe the growth of CNP in your county.

Program Implementation Questions

4. How does CNP implementation vary between institutions, community programs and foster care?

5. Why has CNP been successful in your country?

6. In your opinion, what factors are critical in terms of organizational, process, technical factors?

7. Do you think CNP be simplified without undermining its effectiveness? Which elements of CNP are essential?

8. How have you adapted the program to specifically to your country/ programs?

9. Is there anything special or unique about the social or political context, or general circumstances of the program in your country that would need to be present for CNP to be successfully implemented or replicated? (e.g., cultural, ethnic, or religious values/characteristics; distribution of power; homogeneity; economic conditions)

Program Growth and Expansion

10. Do you think your country program have the desire and organizational capacity to expand its operations and deliver services on a substantially larger scale? If yes/ no, explain.

11. Should the scaling up effort include policy change by the government or rely exclusively on voluntary adoption of the program by private or non-governmental organizations?

12. Do relevant stakeholders, potential partners, and intended beneficiaries perceive a need for this kind of program?

13. As the program grows, what could CNP do to maintain its effectiveness?

14. Are there any procedures for documenting the progress, lessons learned, and implementation of CNP to inform growth?

15. How can program characteristics that were key to the outcomes achieved be replicated or enlarged? (ToT, more training opportunities)

16. What is your vision for CNP in your country? Does the plan include a clear description of proposed actions, timetables, roles, responsibilities, and resources available?

17. How can we best achieve buy-in from the leadership and staff at potential implementing organizations?

18. What additional human, institutional, and financial resources will be needed to support the process of expanding CNP in your country?

19. What human, institutional, and financial resources will be needed for operating at a bigger scale?

20. What new partnerships will need to be established to grow CNP, if any?

21. What success factors do you think need to be in place at sites expected to implement CNP? Can you expand on this?

22. Are action plans and budgets in place for growth of CNP? If not, what more needs to be done?

23. What are the most effective networks and alliances for carrying out advocacy for the growth of CNP? How can they be most efficiently mobilized and organized?

Closing

24. Overall, what are some of the biggest challenges faced by your organization (KBF/ Holt Mongolia) in daily work? Why are they challenges?

25. Is there anything else you would like to tell me about the Child Nutrition Program?

26. Is there other information you think is important for us to know?
